# GSKJ4 Protects Mice Against Early Sepsis via Reducing Proinflammatory Factors and Up-Regulating MiR-146a

**DOI:** 10.3389/fimmu.2018.02272

**Published:** 2018-10-02

**Authors:** Yuchen Pan, Jiali Wang, Yaxian Xue, Jiaojiao Zhao, Dan Li, Shaolong Zhang, Kuanyu Li, Yayi Hou, Hongye Fan

**Affiliations:** ^1^State Key Laboratory of Pharmaceutical Biotechnology, Division of Immunology, Medical School, Nanjing University, Nanjing, China; ^2^State Key Laboratory of Natural Medicines, School of Life Science and Technology, China Pharmaceutical University, Nanjing, China; ^3^Jiangsu Key Laboratory of Molecular Medicine, Nanjing, China

**Keywords:** GSKJ4, JMJD3, sepsis, miR-146a, inflammation

## Abstract

Sepsis, defined as life-threatening organ dysfunction, is one of the most common causes of mortality in intensive care units with limited therapeutic options. However, the mechanism underlying the regulation of epigenetics on sepsis remains largely undefined. Here we showed that JMJD3, the histone lysine demethylase, played a critical role in the epigenetic regulation of innate immunity during early sepsis. Pharmacological inhibition of JMJD3 by GSKJ4 protected mice against early septic death and reduced pro-inflammatory cytokine interleukin-1β (IL-1β) production as well as IL-6, tumor necrosis factor-α (TNF-α), and monocyte chemotactic protein-1 (MCP-1) expression. Interestingly, GSKJ4 up-regulated the transcription of anti-inflammatory microRNA-146a (miR-146a) in peritoneal macrophages from septic mice. Mechanistically, JMJD3 negatively regulated the transcription of miR-146a via its demethylation of H3K27me3 on the promoter of miR-146a. Moreover, the transcription of miR-146a was positively regulated by nuclear factor-κB (NF-κB) p65. Inhibition of NF-κB p65 promoted JMJD3 binding to miR-146a promoter and decreased the tri-methylation level of H3K27, while the inhibition of JMJD3 did not affect the recruitment of NF-κB p65 to miR-146a promoter. These results highlight an epigenetic mechanism by which JMJD3 was inhibited by NF-κB p65 from binding to miR-146a promoter to promote the anti-inflammatory response. Taken together, our findings uncover a key role for JMJD3 in modulating the miR-146a transcription and shed light on the JMJD3 inhibitors could be potential therapeutic agents for early sepsis therapy.

## Introduction

Sepsis is a heterogeneous syndrome that usually characterized as life-threatening organ dysfunction caused by infection (1). During pathogen infection, host innate immune system is strongly activated by pathogen-associated molecular patterns (PAMPs) to release interleukin-1β (IL-1β) and other pro-inflammatory cytokines. The cytokine storm can exacerbate inflammatory responses in the host, which leads to multiple organ failure and even early septic mortality (2, 3). Targeted inhibition of key inflammatory regulators that control dysregulated immune response could be a useful strategy for early sepsis therapy.

Pathogen-mediated epigenetic regulation of gene expression plays an essential role in regulating innate immune cell function in patients with sepsis (4, 5). Especially, the endotoxin lipopolysaccharide (LPS) can epigenetically control the inflammatory response, which contributes to the morbidity and mortality of gram-negative sepsis. LPS stimulation increases transcription of histone lysine demethylase JMJD3 through nuclear factor-κB (NF-κB) activation in macrophages (6). JMJD3 specifically relies on its demethylation of di- and tri-methyl-lysine 27 on histone H3 (H3K27me2/3) to promote the transcription of inflammatory cytokines. In early sepsis, JMJD3 contributes to the production of pro-inflammatory IL-1β (7). Inhibition of JMJD3 by GSKJ4, a small-molecule inhibitor of JMJD3, decreases the expression of inflammatory mediators IL-1β, IL-6, tumor necrosis factor-α (TNF-α), etc. (8–10). However, the therapeutic effect of GSKJ4 on sepsis is unknown.

MicroRNAs (miRNAs) is involved in post-transcriptional regulation, and mediates protein expression by targeting specific mRNAs degradation. A number of miRNAs have been shown to regulate LPS-induced inflammatory cascade (11, 12). For example, miRNA-146a (miR-146a) is positively regulated by NF-κB p65 under LPS stimulation (13). MiR-146a also acts as a negative feed-back regulator of NF-κB p65 by targeting MyD88 and TRIF degradation, resulting in reduction of inflammatory cytokines (14, 15). Moreover, miR-146a can also inhibit JMJD3 expression through its interaction with the 3′UTR of JMJD3 (16). Therefore, miR-146a is considered to be an effective anti-inflammatory regulator with multiple targets. Moreover, plasma/serum miR-146a level from sepsis patients is slightly decreased compared with non-sepsis-SIRS samples (17, 18). Interestingly, exosomal miR-146a contributes to the enhanced therapeutic efficacy of IL-1β-primed mesenchymal stem cells against sepsis (19). Despite substantial investigation on the anti-inflammatory effect of miR-146a, other key transcriptional factors that regulate miR-146a transcription remain unclear.

In this study, the protective effect of GSKJ4 against sepsis from inflammation was investigated in a clinical *Escherichia coli*-induced sepsis model. We found that GSKJ4 treatment protected sepsis against inflammation. GSKJ4 also up-regulated miR-146a expression in peritoneal macrophages from sepsis mice. Interestingly, we provide evidence to support a critical role of JMJD3 in the negative regulation of miR-146a transcription in macrophages. Our data supplemented that how host innate immunity was epigenetically regulated by JMJD3, and suggested that JMJD3 inhibitors could be potential therapeutic agents for early sepsis therapy.

## Results

### GSKJ4 protects *Escherichia coli*-induced sepsis against inflammation and up-regulates miR-146a expression

**Figure 1:**
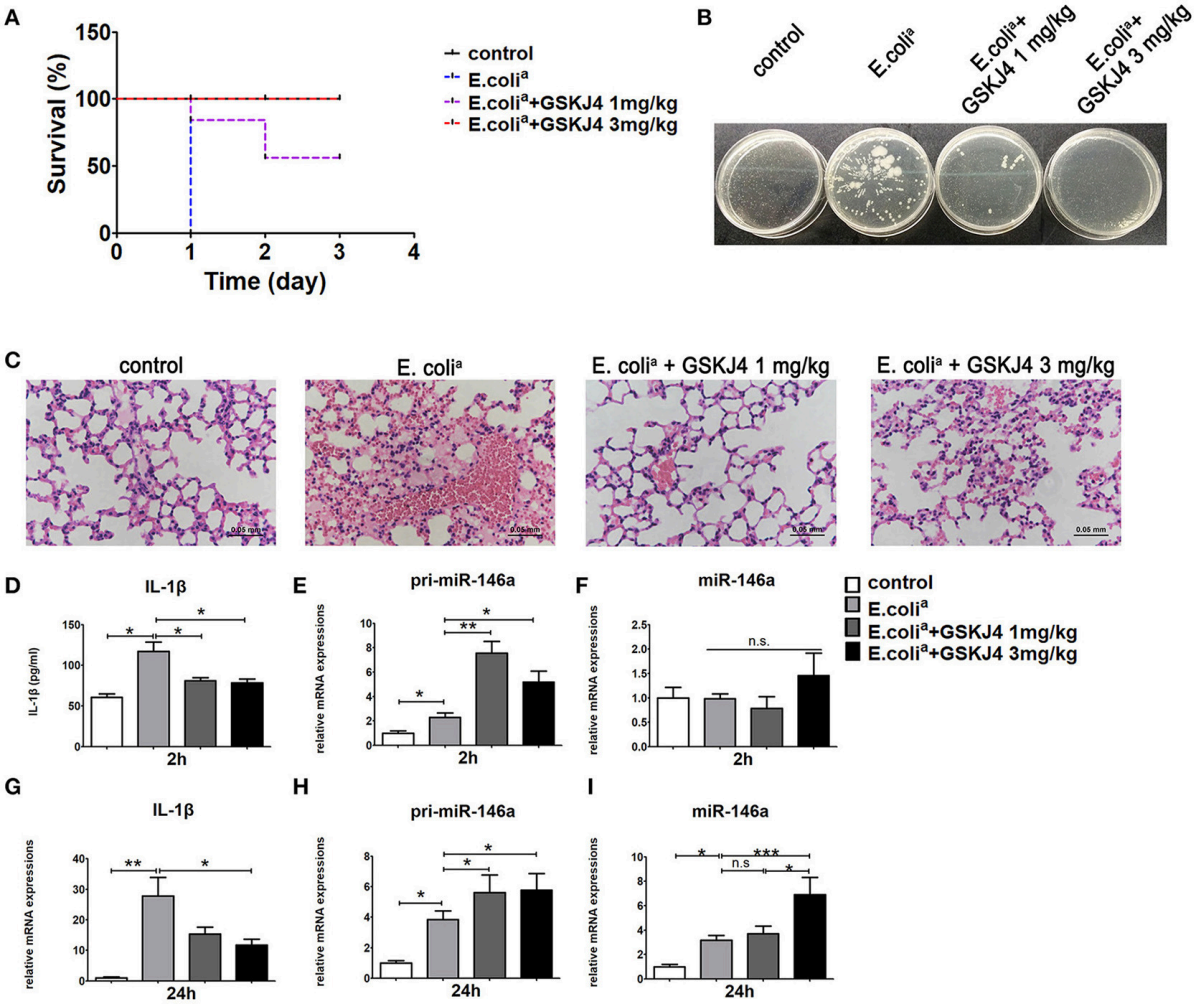
GSKJ4 protects mice against septic death. ICR mice were administered intraperitoneally (i.p.) with GSKJ4 (1 or 3 mg/kg body weight) or normal saline 1 h prior to bacterial infection intraperitoneally (i.p.) with viable clinical *Escherichia coli* (*E. coli*^*a*^) (5 × 10^7^ CFU/mouse). **(A)** Mouse survival was monitored every day. Survival curves were used to analyze the data (7 mice per group). **(B–I)** Mice were treated as in **(A)** except that mice were injected (i.p.) with *E. coli*^*a*^ (1 × 10^7^ CFU/mouse). **(B)** Blood from mice 24 h after infection was plated for 16 h. Representative plate shows bacterial colonies. **(C)** Representative Hematoxylin and eosin (H&E) stain of lung sections of mice 24 h after infection. **(D)** The serum level of IL-1β at 2 h post bacterial infection was measured by ELISA kit (7 mice per group). The mRNA expression levels of pri-miR-146a **(E,H)**, miR-146a **(F,I)**, and IL-1β **(G)** in peritoneal macrophage were determined by quantitative PCR (7 mice per group). Data are shown as mean ± SEM (*n* = 7). ^*^P < 0.05; ^**^P < 0.01; ^***^P < 0.001.

To confirm the anti-inflammatory effect of GSKJ4 *in vivo*, we used well-established sepsis model in ICR mice. The clinical *Escherichia coli* (*E. coli*^*a*^ for short) strains were isolated from human clinical specimens and injected intraperitoneally at the concentration of 5 × 10^7^ CFU per mouse. The 24 h survival rate of septic mice was 0%, while low dose of GSKJ4 (1 mg/kg) treatment increased the survival rate to 50%, and high dose of GSKJ4 (3 mg/kg) treatment improved survival to 100% (Figure 1A). To further investigate the changes of inflammatory factors in all groups of mice, we used different concentrations of *E. coli*^*a*^ to establish sepsis model during preliminary experiment. We found that 1 × 10^7^ CFU per mouse was the highest concentration where all mice in model group could be alive up to 24 h. Mice infected with *E. coli*^*a*^ (1 × 10^7^ CFU/mouse) were sacrificed at 24 h, peripheral blood was collected and the level of bacterial burden was assessed by cloning assay (20). The potential of GSKJ4 for synergistic bactericidal activity was also evaluated by static time-kill experiment at different concentrations using inocula of 1 × 10^5^ CFU/mL. GSKJ4 had no effect on the growth of *E. coli*^*a*^ *in vitro* (Supplementary Figure 1). Compared with septic mice, the bacterial CFU counts in the blood of GSKJ4 treated mice were significantly decreased (Figure 1B). Furthermore, septic mice had serious congestion in the alveolar walls with severe inflammatory cell infiltration. GSKJ4 treatment remarkably decreased inflammatory cell infiltration and hyperemia in the alveolar walls (Figure 1C). The peripheral serum IL-1β was apparently rising up within 2 h in septic mice, while GSKJ4 treatment reduced serum IL-1β levels (Figure 1D).

To determine the transcriptional pattern of *miR-146a* in septic mice, peritoneal macrophages from mice of all groups were collected at 2 and 24 h. The expression of *pri-miR-146a* was up-regulated in septic mice at 2 and 24 h, while its up-regulation was further enhanced by GSKJ4 treatment (Figures 1E,H). The expression of mature *miR-146a* was unchanged at 2 h but up-regulated at 24 h (Figures 1F,I). Moreover, GSKJ4 treatment down-regulated the transcription of *IL-1*β at 24h (Figure 1G). In addition, the expressions of *TNF-*α, *IL-6*, and *MCP-1* were elevated in septic mice. Their expressions were unchanged at 2 h under GSKJ4 treatment, while down-regulated at 24 h (Supplementary Figure 2). Results above indicated that GSKJ4 could improve the survival of sepsis mice and inhibit the inflammation induced by *E. coli*^*a*^.

**Figure 2:**
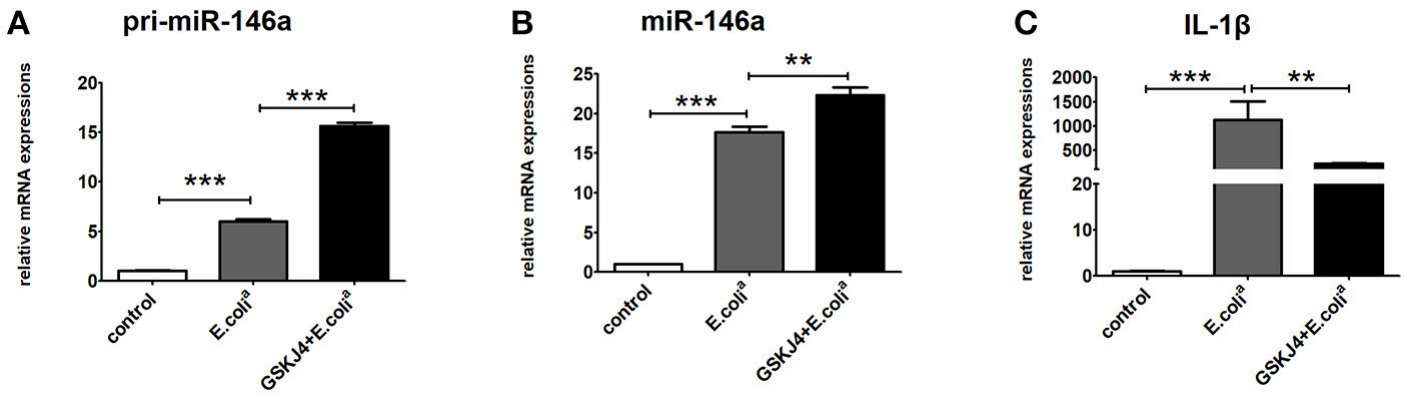
GSKJ4 amplifies miR-146a expression under *Escherichia coli (E. coli*^*a*^*)*-stimulation in macrophages. Raw264.7 cells were pre-treated with or without GSKJ4 (4 μmol/L) for 1 h, followed by stimulation with *E. coli*^*a*^ (10^7^ CFU/mL) for 24 h. The mRNA expression levels of pri-miR-146a **(A)**, miR-146a **(B)**, and IL-1β **(C)** in Raw264.7 were determined by quantitative PCR. Data are shown as mean ± SEM (*n* = 3). ^**^P < 0.01; ^***^P < 0.001.

To verify whether GSKJ4 affects *miR-146a* expression *in vitro*, Raw264.7 cells were pre-treated with GSKJ4 and then stimulated with *E. coli*^*a*^ for 24 h. Compared with *E. coli*^*a*^ stimulation, GSKJ4 treatment significantly enhanced the transcription of both *pri-miR-146a* and *miR-146a*, but down-regulated *IL-1*β induced by *E. coli*^*a*^ (Figures 2A–C).

### miR-146a transcription pattern in LPS-stimulated macrophages

**Figure 3:**
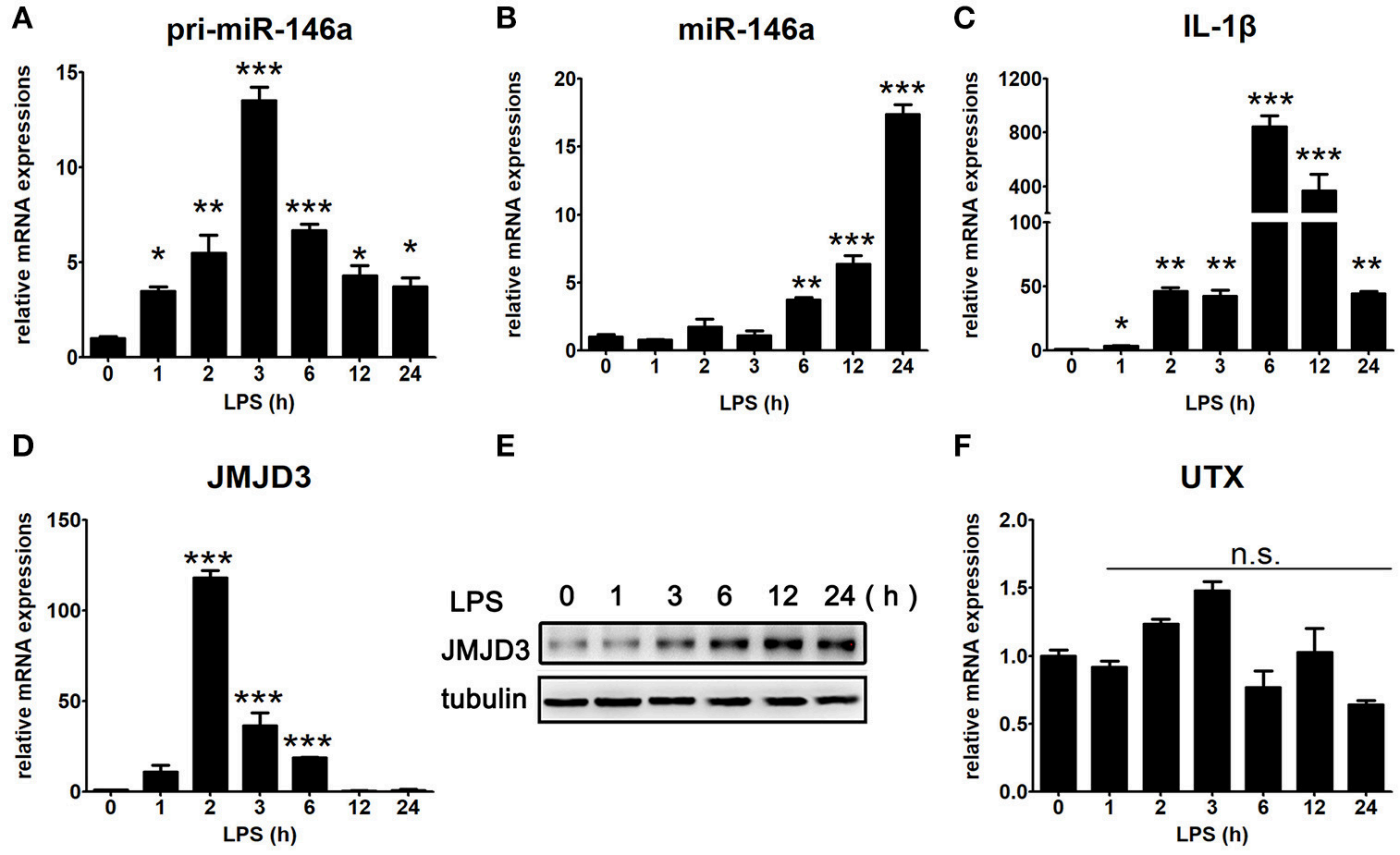
Time specific expression pattern of inflammatory factors under LPS stimulation in macrophages. Raw264.7 cells were treated with LPS (100 ng/mL). The mRNA expression levels of pri-miR-146a **(A)**, miR-146a **(B)**, IL-1β **(C)**, JMJD3 **(D)**, and UTX **(F)** in Raw264.7 were determined by quantitative PCR. **(E)** The protein expression level of JMJD3 was determined by Western blotting. Data are shown as mean ± SEM (*n* = 3). ^*^P < 0.05; ^**^P < 0.01; ^***^P < 0.001.

LPS is considered to be responsible for the clinical manifestations of septic inflammation (21). To investigate the time point at which miR-146a starts to transcript and mature, LPS-stimulated Raw264.7 cells were collected at different time intervals for up to 24 h. Interestingly, the transcription of *pri-miR-146a* was started almost immediately after LPS-stimulation (Figure 3A), while mature *miR-146a* increased drastically from 6 h to last for 24 h in a time-dependent manner (Figure 3B). Moreover, *IL-1*β quickly started its transcription as early as 1 h and reached to peak expression level at 6 h, and then gradually deceased (Figure 3C). Furthermore, JMJD3, a histone demethylase, quickly peaked its transcription level at 2 h and declined to normal level at 12 h (Figure 3D), while it sustained high protein expression level for at least 24 h (Figure 3E). Of note, the transcription of *UTX*, a homologous gene closely related to JMJD3, was relatively stable under LPS stimulation (Figure 3F). It is known that NF-κB can be activated by LPS within 10 min, and the transcription of *miR-146a, IL-1*β, and *JMJD3* are all promoted by NF-κB activation. We speculate that the transcription of miR-146a may be co-regulated by some other proteins, yet the information about relative transcription factors is limited.

### GSKJ4 promotes transcription of miR-146a and decreases the levels of pro-inflammatory cytokines

**Figure 4:**
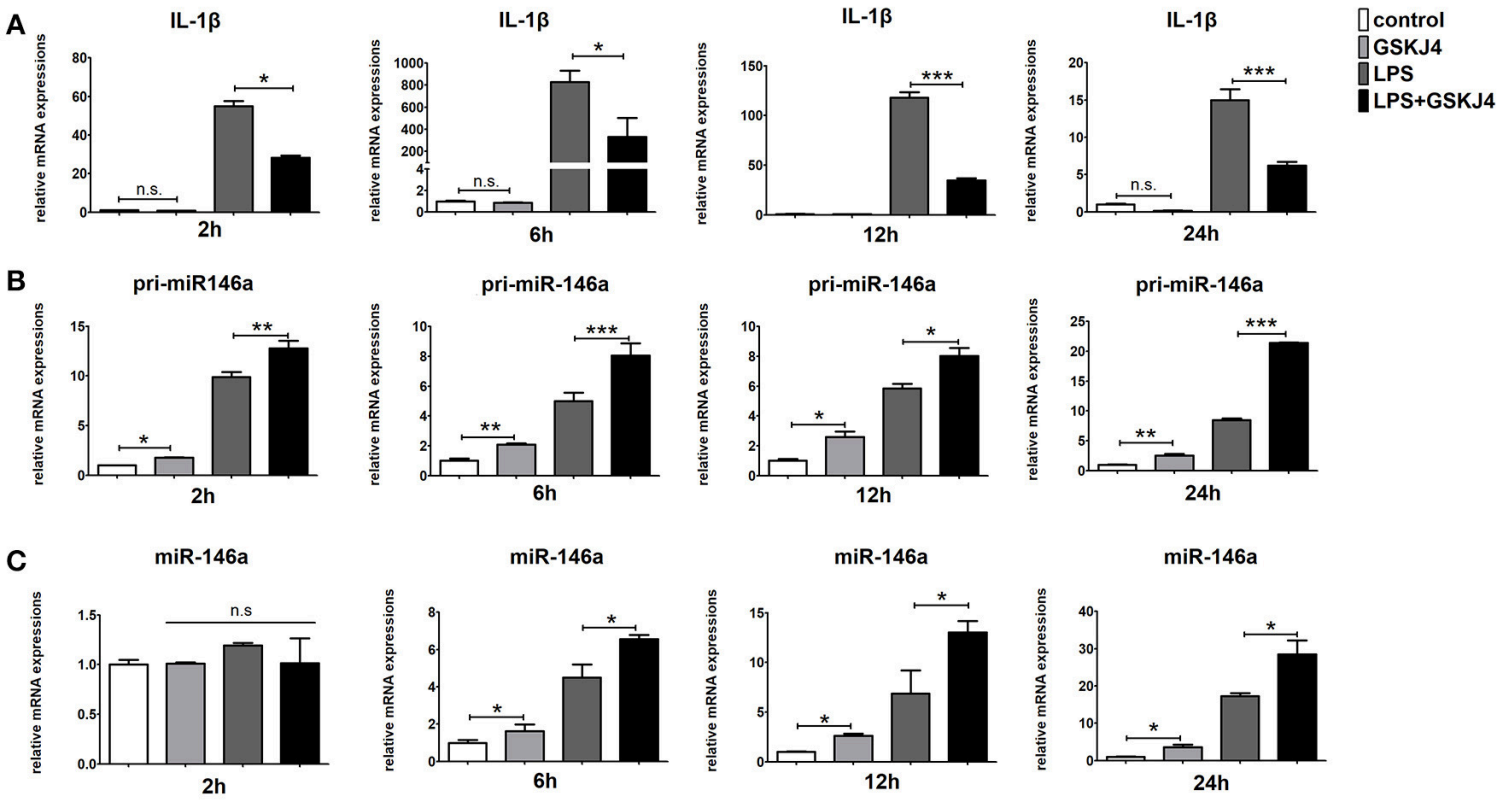
GSKJ4 amplifies miR-146a expression under LPS-stimulation in macrophages. Raw264.7 cells were pre-treated with or without GSKJ4 (4 μmol/L) for 1 h, followed by stimulation with LPS (100 ng/mL). The mRNA expression levels of IL-1β **(A)**, pri-miR-146a **(B)**, and miR-146a **(C)** in Raw264.7 were determined by quantitative PCR at different time. Data are shown as mean ± SEM (*n* = 3). ^*^P < 0.05; ^**^P < 0.01; ^***^P < 0.001.

**Figure 5:**
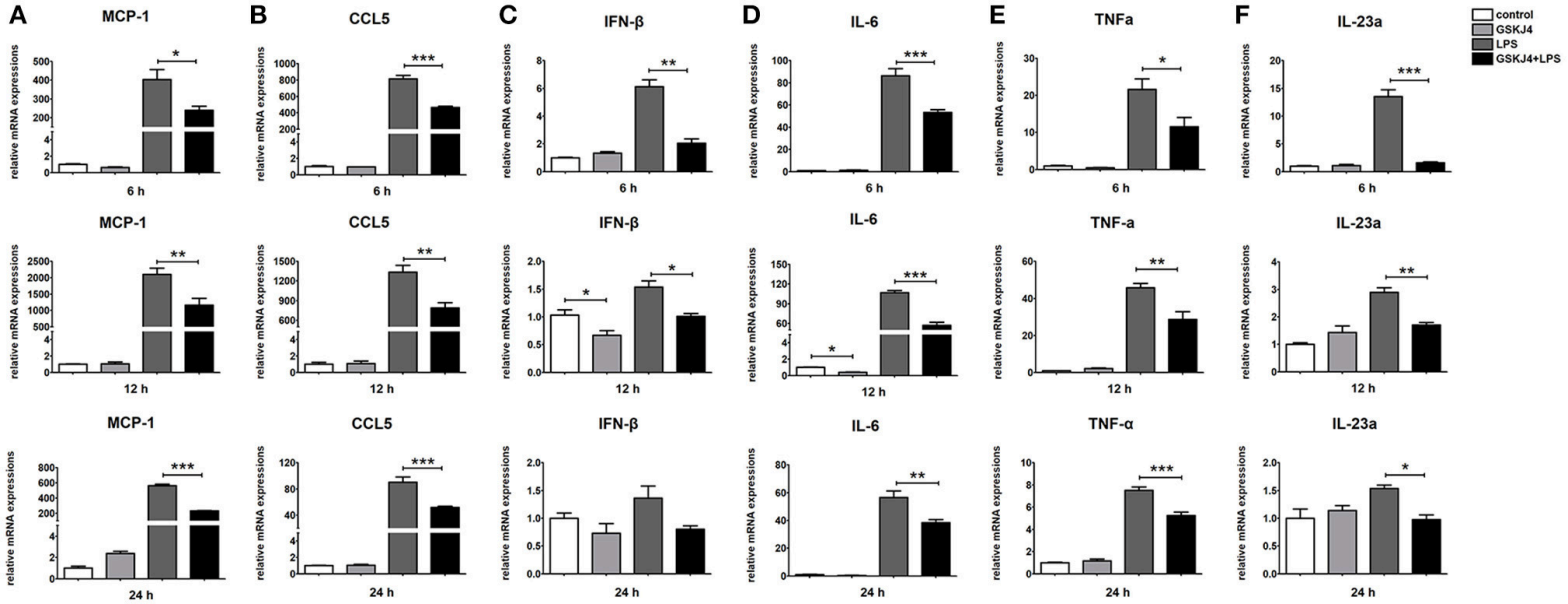
GSKJ4 suppresses the expressions of inflammatory cytokines under LPS-stimulation in macrophages. Raw264.7 cells were pre-treated with or without GSKJ4 (4 μmol/L) for 1 h, followed by stimulation with LPS (100 ng/mL). The mRNA expression levels of MCP-1 **(A)**, CCL5 **(B)**, IFN-β **(C)**, IL-6 **(D)**, TNF-α **(E)**, and IL-23a **(F)** in Raw264.7 were determined by quantitative PCR at different time. Data are shown as mean ± SEM (*n* = 3). ^*^P < 0.05; ^**^P < 0.01; ^***^P < 0.001.

To determine whether JMJD3 influences the transcription of *miR-146a*, Raw264.7 cells with or without LPS-stimulation were pre-treated with GSKJ4. Inhibition of JMJD3 by GSKJ4 at 4 μmol/L significantly decreased *IL-1*β transcription in LPS-stimulated Raw264.7 cells (Figure 4A), but did not affect the cell viability (Supplementary Figure 3). Of note, GSKJ4 treatment up-regulated the transcription of *pri-miR-146a* in Raw264.7 cells with or without LPS-stimulation as early as 2 h and lasted for at least 24 h (Figure 4B). And the expression of *miR-146a* was significantly up-regulated 6 h after GSKJ4 pre-treatment and lasted for at least 24 h (Figure 4C). Moreover, GSKJ4 treatment decreased the expression of *MCP-1, CCL-5*, and *IFN-*β, that downstream to miR-146a, as well as *IL-6, TNF-*α, and *IL-23a*, that downstream to JMJD3 (Figures 5A–F). Taken together, these results suggested that JMJD3 is involved in the regulation of *miR-146a* transcription.

### Binding of JMJD3 at regulatory sequences causes transcriptional inhibition of miR-146a in macrophages

**Figure 6:**
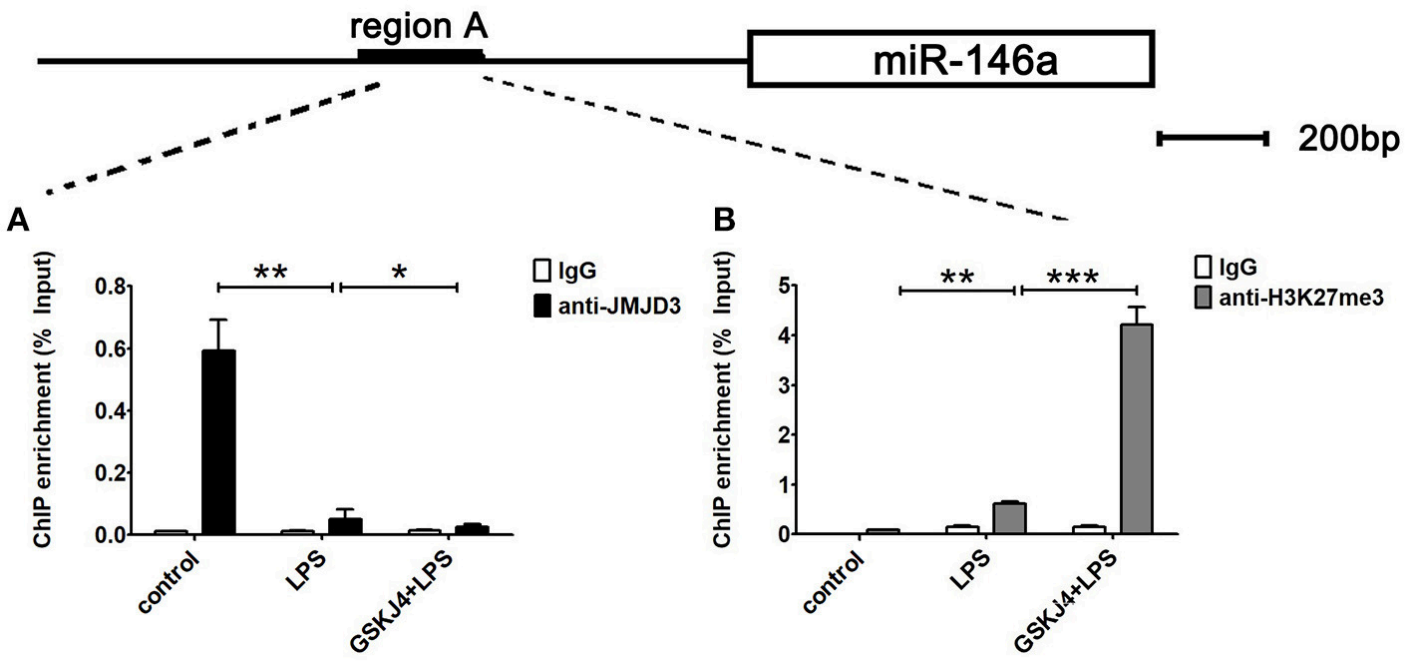
GSKJ4 restrains the recruitment of JMJD3 to the promoter region of miR-146a. Raw264.7 cells were pre-treated with or without GSKJ4 (4 μmol/L) for 1 h, followed by stimulation with LPS (100 ng/mL) for 24 h. **(A,B)** ChIP assay was performed using anti-JMJD3, anti-trimethyl H3K27 (H3K27me3), and IgG. The occupancy of each protein was determined with quantitative PCR at the gene promoter region encompassing the JMJD3 binding site. ChIP assay using normal IgG was performed as a negative control. Data are shown as mean ± SEM (*n* = 3). ^*^P < 0.05; ^**^P < 0.01; ^***^P < 0.001.

To examine whether *miR-146a* is transcriptionally regulated by JMJD3, ChIP assays were performed to detect the direct binding site of JMJD3 to *miR-146a* promoter. Isolated chromatin from Raw264.7 cells was immunoprecipitated with either anti-JMJD3 antibody or corresponding negative antibody, followed by quantitative PCR analysis with primers targeted to sequences on the upstream of *miR-146a*. As shown in Figure 5A, JMJD3 could physically bind to regulatory sequences on the upstream of *miR-146a* in region A, ranging from 1,942 to 2,173 bp. However, the recruitment of JMJD3 was decreased in LPS-stimulated Raw264.7 cells, and the recruitment declined more with GSKJ4 pre-treatment (Figure 6A). Moreover, since it was reported that JMJD3 relied on its demethylase activity to regulate gene transcription, the effect of JMJD3 on the tri-methylated H3K27 level on the *miR-146a* promoter has to be investigated. Isolated chromatin from Raw264.7 cells was immunoprecipitated with either anti-H3K27me3 antibody or corresponding negative antibody. ChIP assays showed an increased level of tri-methylated H3K27 (H3K27me3) at the miR-146a promoter in LPS-stimulated Raw264.7 cells, and the level was mounting more with GSKJ4 pre-treatment (Figure 6B). These results above indicated that JMJD3 negatively regulated the transcription of *miR-146a* via its demethylation of H3K27me3 on the promoter of *miR-146a*.

### NF-κB p65 inhibits JMJD3 binding to miR-146a promoter

**Figure 7:**
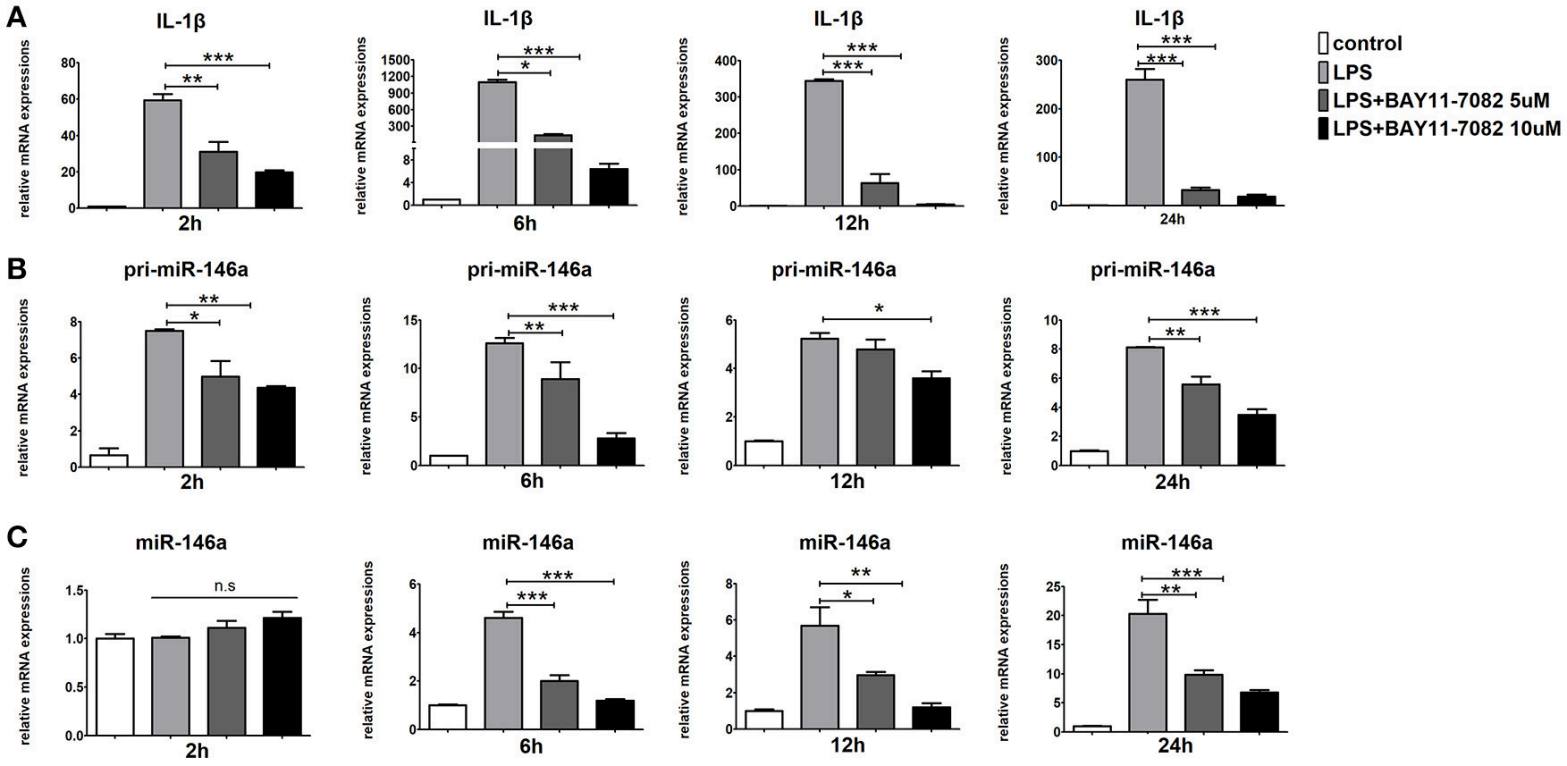
Inhibition of NF-κB blocks miR-146a transcription in macrophages. Raw264.7 cells were pre-treated with or without BAY11-7082 (5 μmol/L or 10 μmol/L) for 1 h, followed by stimulation with LPS (100 ng/mL). The mRNA expression levels of IL-1β **(A)**, pri-miR-146a **(B)**, and miR-146a **(C)** in Raw264.7 were determined by quantitative PCR at different time. Data are shown as mean ± SEM (*n* = 3). ^*^P < 0.05; ^**^P < 0.01; ^***^P < 0.001.

It has been reported that NF-κB p65 are responsible for the transcription of *miR-146a*. As expected, BAY11-7082, the NF-κB inhibitor, strongly down-regulated *pri-miR-146a, miR-146a*, and *IL-1*β expression in LPS-stimulated Raw264.7 cells (Figures 7A–C). These results implied that the transcription of *miR-146a* was positively regulated by NF-κB.

**Figure 8:**
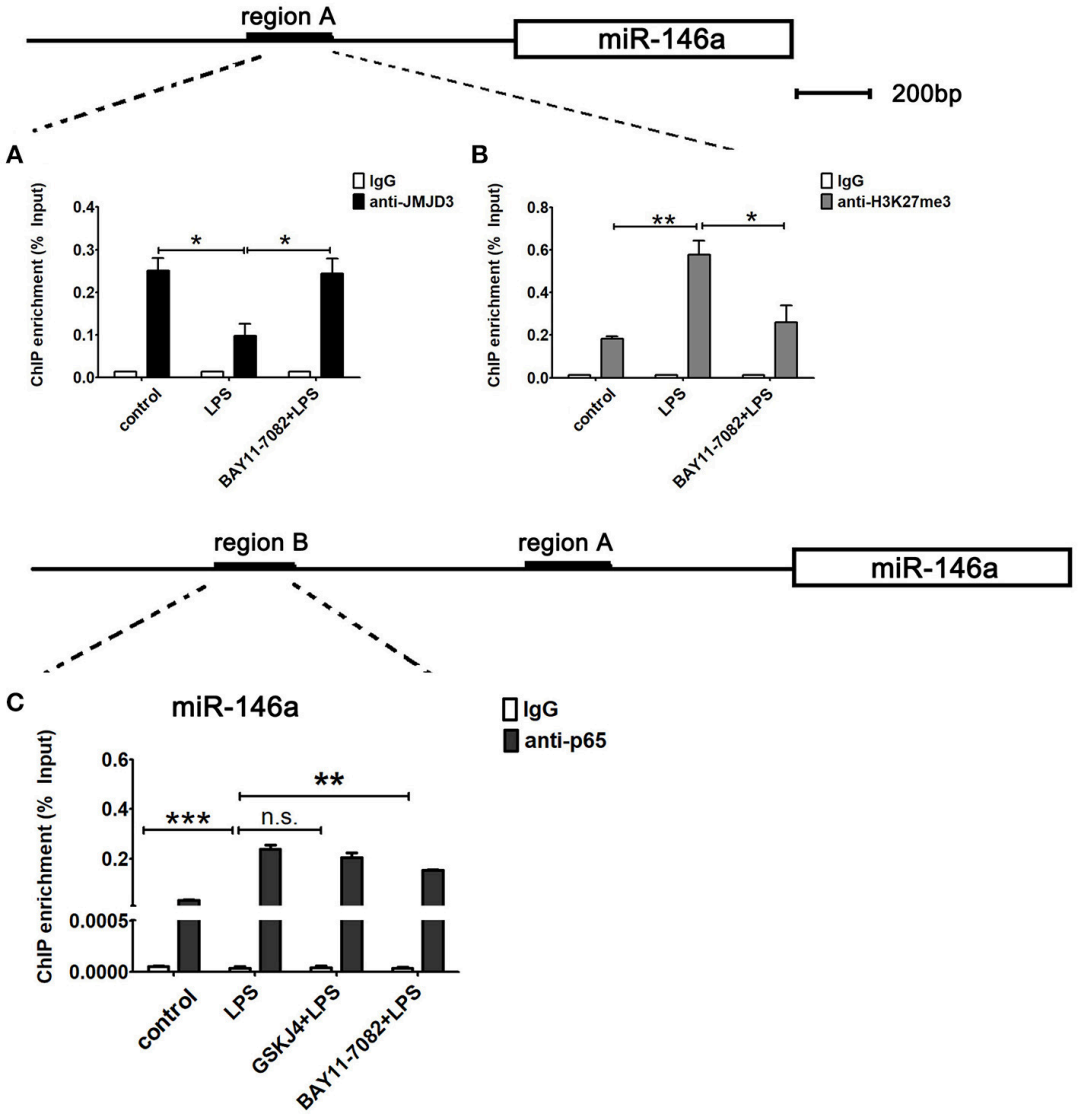
miR-146a is transcriptionally co-regulated by JMJD3 and NF-κB p65. Raw264.7 cells were pre-treated with GSKJ4 (4 μmol/L) or BAY11-7082 (5 μmol/L) for 1 h, followed by stimulation with LPS (100 ng/mL) for 24 h. **(A–C)** ChIP assay was performed using anti-JMJD3, anti-trimethyl H3K27 (H3K27me3), anti-p65, and IgG. The occupancy of each protein was determined with quantitative PCR at the gene promoter region encompassing the JMJD3 or NF-κB binding site. ChIP assay using normal IgG was performed as a negative control. Data are shown as mean ± SEM (*n* = 3). ^*^P < 0.05; ^**^P < 0.01; ^***^P < 0.001.

Next, to examine whether *miR-146a* is transcriptionally co-regulated by JMJD3 and NF-κB p65, ChIP assays were performed to detect the binding of JMJD3, H3K27me3, and p65 to the promoter of *miR-146a* in LPS-stimulated Raw264.7 cells under BAY11-7082 pre-treatment. LPS stimulation decreased the recruitment of JMJD3 on *miR-146a* promoter, which was significantly recovered by BAY11-7082 pre-treatment (Figure 8A). Correspondingly, the increased tri-methylation level of H3K27 on *miR-146a* promoter under LPS stimulation was down-regulated by BAY11-7082 pre-treatment (Figure 8B). These results indicated that inhibition of NF-κB p65 promoted JMJD3 binding to *miR-146a* promoter and changed the tri-methylation level of H3K27. Furthermore, isolated chromatin from Raw264.7 cells was immunoprecipitated with either anti-p65 antibody or corresponding negative antibody, and followed by quantitative PCR analysis with primers targeted to sequences in region B on the upstream of *miR-146a*. LPS stimulation facilitated p65 binding to the region B on miR-146a promoter, while GSKJ4 pre-treatment did not affect the recruitment of p65. On the contrary, BAY 11-7082 pre-treatment decreased the recruitment of p65 to the *miR-146a* promoter upon LPS stimulation (Figure 8C). Thus, these results indicated that NF-κB p65 inhibited JMJD3 binding to *miR-146a* promoter.

## Discussion

The innate immune system is the primary line of defense against the invasion of pathogens. However, it could cause dysregulated inflammatory immune network when innate immune cells became over-activated, leading to sepsis and ultimately death. Studies have demonstrated that epigenetic regulator plays an essential role in the negative regulation of the inflammatory factors. The elucidation of epigenetic regulation on anti-inflammatory factors can shed light on therapeutic strategies for early sepsis therapy. Here, we provide evidence supporting JMJD3 as an epigenetic regulator to contribute to the regulation of the miR146a transcription and the excessive inflammatory response in microbial sepsis. Pharmacological inhibition of JMJD3 relieved over-activated innate immunity to infection and rendered mice more resistant to early sepsis mortality.

JMJD3, a key enzyme for demethylation of H3K27, increases immune responses and elicits inflammation. JMJD3 can be quickly and strongly induced by the transcription factor NF-κB in macrophages exposed to bacterial products and inflammatory cytokines (6), while many of LPS inducible inflammatory genes were found to be Jmjd3 targets. Importantly, JMJD3 fine-tunes the transcriptional output of LPS-activated macrophages in an H3K27 demethylation dependent or independent manner (22–24). Moreover, JMJD3 epigeneticly regulates Th17 cell differentiation by directly bound to and reduced the level of H3K27me3 at the genomic sites of *Rorc*, which ecodes the major Th17 transcription factor Rorγt (25). Therefore, pharmacological inhibition of Jmjd3 is assumed to be a novel therapeutic strategy for suppressing autoimmune responses. GSKJ4, a JMJD3 selective inhibitor, decreases pro-inflammatory factor production via inhibition of JMJD3 and promotion of cellular H3K27 methylation. Administration of GSKJ4 ameliorates the severity of experimental autoimmune encephalomyelitis (EAE) *in vivo*, and limits inflammation through the induction of a tolerogenic phenotype on DCs (8). In addition, GSKJ4 has shown great therapeutic effect on T cell acute lymphoblastic leukemia (T-ALL) (26) and pediatric brainstem glioma (27). Despite this, GSKJ4 could also affect macrophage recruitment and extravasation. Therefore, GSKJ4 is considered to have potential therapeutic effects on inflammation induced diseases and merits further investigation.

Excessive or poorly controlled pro-inflammatory cytokines leads to multiple organ failure and even early sepsis mortality. Plasma cytokine levels of IL-1β, TNF-α, IL-6, and MCP-1 were markedly increased in patients with sepsis compared to those in healthy control subjects. Interestingly, JMJD3 expression levels in neutrophils were increased and contributes to the production of pro-inflammatory IL-1β in early sepsis (7). In this study, we showed that GSKJ4 protects mice against septic death and reduces the expression of inflammatory factors IL-1β, TNF-α, IL-6, and MCP-1 *in vivo*, suggesting that JMJD3 may be the key molecule driving early sepsis progression. Moreover, the efficiency of bacterial clearance is essential in limiting sepsis-induced inflammation and organ injury (28). We confirmed that GSKJ4 promoted bacterial clearance in the peripheral blood, suggesting that GSKJ4 may be involved in enhancing phagocytosis that helps to limit inflammation.

MicroRNAs can correct excessive host response to infection in an epigenetic regulatory way. Especially, miR-146a was identified as a negative regulator of TLR/NF-κB-mediated innate immune and inflammatory responses (29). Clinically, several studies indicated that miR146a has the potential to serve as new biomarkers for sepsis, and miR146a level in serum, plasma, and whole blood were lower in sepsis patients (17, 18, 30). Moreover, *in vivo* studies showed that Lentivirus-expressing miR-146a transfection attenuated sepsis-induced inflammatory cytokine production in both plasma and peritoneal fluid (31). However, the connection between decreased miR146a and poor outcome of sepsis remains unexplained. Here, our study showed that GSKJ4 treatment up-regulated the transcription of anti-inflammatory miR146a in peritoneal macrophages from septic mice. *In vitro* experiments also showed that GSKJ4 promotes miR146a transcription, suggesting that JMJD3 is involved in the regulation of miR146a transcription. Furthermore, we observed that JMJD3 can physically bind to the promoter of miR-146a. The recruitment of JMJD3 on miR-146a promoter was declined more with GSKJ4 pre-treatment in LPS-stimulated Raw264.7 cells and accompanied with the increased level of H3K27me3, suggesting that JMJD3 negatively regulates the transcription of miR146a depending on its demethylation of H3K27me3. Interestingly, the transcription of miR146a is positively regulated by the activation of NF-κB p65, so there is a need to investigate how JMJD3 and NF-κB p65 co-regulate miR-146a transcription. We observed that LPS stimulation facilitated p65 binding to miR-146a promoter, while GSKJ4 pre-treatment did not affect the recruitment of p65, suggesting that inhibition of JMJD3 did not affect NF-κB p65 binding to miR146a promoter. However, inhibition of NF-κB p65 promoted JMJD3 binding to miR146a promoter and decreased the level of H3K27me3, demonstrating that the activation of NF-κB p65 inhibits JMJD3 from binding to miR-146a promoter, which facilitates the transcription of miR146a.

Numerous studies confirmed the dominant anti-inflammatory effect of miR-146a. *In vitro* studies showed that over-expression of miR146a decreased expression of inflammatory genes induced by IL-1β, while inhibition of miR146a increased the production of IL-1β and MCP-1. In addition, miR146a negatively regulated IL-1 receptor-associated kinase 1 (IRAK1) via binding to its 3′-UPR, and negatively regulated IL-1β, IL-6 and TNF-α secretion consequently in human gingival fibroblasts following LPS-stimulation (32). As a matter of fact, excessive inflammatory factors were rapidly produced by bacteria or LPS infection in early sepsis, while the anti-inflammatory factors were usually undetectable at early stage of infection. As suggested by our findings *in vivo*, the expression of inflammatory factors IL-1β, TNF-α, IL-6, and MCP-1 were significantly up-regulated in peritoneal macrophage from septic mice at 2 h, surprisingly pri-miR146a was also up-regulated at 2 h while mature miR146a were increased at 24 h. Furthermore, our study showed that mature miR146a was significantly up-regulated until 6 h after LPS stimulation and reached peak at 24 h *in vitro*, however the transcription of pri-miR146a started as early as 2 h and maintained its expression level unchanged at 24 h. Clinically, miR146a were detected at a lower level in the serum, plasma, and whole blood from sepsis patients (17, 18, 30). But this study did not clarify whether all patients enrolled were required to provide a blood sample within 24 h of the diagnosis of sepsis. Thus, we hypothesize that miR146a expression pattern may differ at different stages of sepsis.

**Figure 9:**
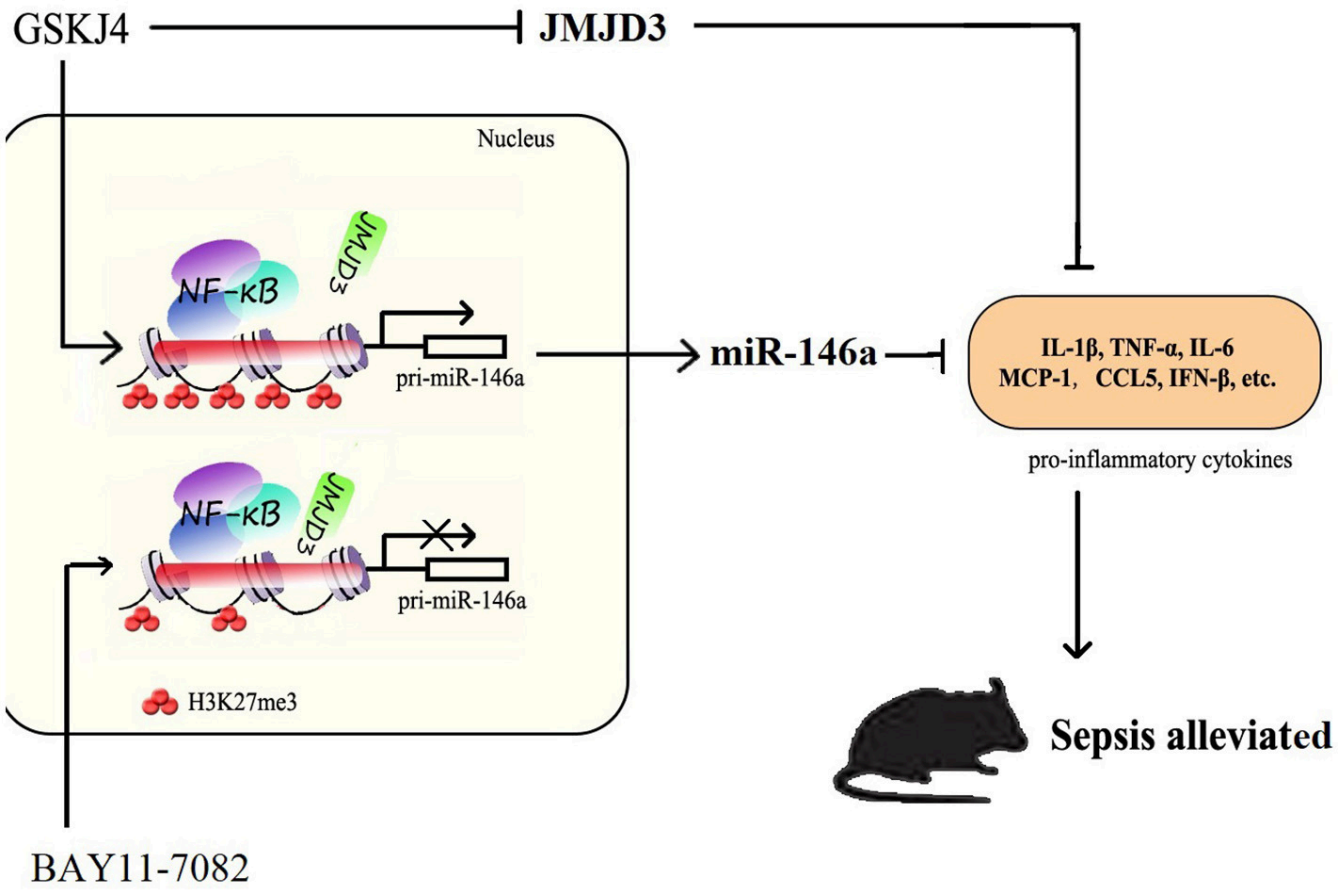
JMJD3 specifically regulates miR-146a expression via its demethylation of H3K27me3. GSKJ4 treatment suppressed the binding of JMJD3 to the promoter region of pri-miR-146a, caused transcription of pri-miR-146a, and then induced the increase of miR-146a. GSKJ4 treatment decreased MCP-1, CCL-5 and IFN-β, that downstream to miR-146a, as well as IL-6, TNF-α and IL-23a, that downstream to JMJD3, ultimately lead to the protective effect against sepsis.

In summary, our studies demonstrate a major role for JMJD3 in regulating the LPS-mediated innate immune response and provided a potential JMJD3-targeting strategy for the clinical therapy of early sepsis. JMJD3 epigenetically inhibited miR-146a transcription depending on its demethylation of H3K27me3. Inhibition of JMJD3 by GSKJ4 led to reduced inflammatory response in sepsis, and up-regulation of miR-146a. These data suggested that JMJD3 inhibitors could be potential therapeutic agents for early sepsis therapy (Figure 9). The underlying molecular mechanism responsible for the negative regulation of JMJD3 to miR-146a awaits further investigation.

## Materials and methods

### Chemicals and ligands

GSKJ4 and LPS (*E. coli* 055:B5) were purchased from Sigma, Shanghai, China. GSKJ4 was prepared in DMSO and LPS was prepared in ddH_2_O. BAY 11-7082 was purchased from Beyotime, China, and prepared in DMSO. The antibodies used for JMJD3 western blot detection were raised in mouse and were obtained from Cell Signaling Technologies (CST; Beverly, MA, USA). The antibody for GAPDH was purchased from Thermo Fisher Scientific (Berlin, Germany). The anti-JMJD3, anti-p65and anti-H3K27me3 antibody were purchased from Cell Signaling Technologies (CST; Beverly, MA, USA).

### Cells and culture conditions

Raw264.7 cells were cultured in DMEM (Gibico, Grand iSland, NY, USA) containing 10% FBS (Gibico), supplemented with penicillin (100 units/ml; Gibco BRL, USA) and streptomycin (100 μg/ml; Gibco BRL, USA), at 37°C in a humidified atmosphere with 5% CO2.

### Bacterial strain

The clinical strains were isolated from human clinical specimens, collected and identified by the Medical Laboratory Center of Zhongda Hospital in Nanjing, Jiangsu, China, and stored at −80°C. Bacterial strains were prepared in beef broth medium.

### Mice

Female ICR mice were purchased from Sino-British SIPPR/BK Lab. Animal Ltd (Shanghai, China). All mice were 5–6 weeks old at time of experimentation. They were acclimatized for 1 week before molding. All procedures involving animals were in strict accordance with protocols approved by the Research Ethics Committee of Nanjing University. All the mice were housed in specific pathogen-free conditions at the Nanjing University Animal Care Commission. *E. coli* isolated from clinical specimens was grown to mid-exponential phase, harvested, washed and resuspended with normal saline (5 × 10^8^ or 1 × 10^8^ CFU/ml). Then 0.1 ml bacterial suspension was injected intraperitoneally (i.p.) into ICR mice (*n* = 7). GSKJ4 (1 or 3 mg/kg) was injected intraperitoneally 1 h before bacterial injection. The animal status and survival rate were recorded daily for up to 3 days. Finally, the animals were humanely terminated, and histological evaluation of lungs was performed. Experiments were repeated three times with each treatment group containing seven mice.

### RNA extraction and quantitative real-time PCR

Total RNA was isolated using Trizol Reagent (Invitrogen) according to the manufacturer's instructions. Real-time PCR assay was then performed using SYBR green dye (Invitrogen) on StepOne sequence detection system (Applied Biosystems, Waltham, MA, USA). Relative abundance of genes was calculated by using 2^−ΔΔCT^ formula, with GAPDH or U6 as an internal control.

### Western blot

Proteins were exacted with an appropriate volume of lysis buffer. After electrophoresis on SDS-PAGE, proteins were transferred onto PVDF membranes (Millipore). Antibodies used here were anti-JMJD3 (Cell Signaling Technology), anti-GAPDH (Cell Signaling Technology), and goat anti-rabbit IgG HRP (Cell Signaling Technology).

### ChIP

ChIP assay was performed according to the protocol of Magna ChIP^TM^ A/G kit (Temecula, CA 92590). Immunoprecipitation of cross-linked chromatin was conducted with anti-JMJD3, anti-H3K27me3 or control mouse monoclonal IgG. After immunoprecipitation, DNA was extracted using the QIAquick PCR purification kit (Qiagen) and an aliquot was amplified by real time PCR. (see Supplementary Methods for primer sequence).

### Statistical analysis

All of the values presented on the graphs are given as means ± S.E.M. ANOVA and unpaired Student's *t*-tests were used to analyze the statistical significance, and *P*-values set to 0.05 were considered statistically significant. GraphPad Prism 5 Demo (GraphPad Software Inc., La Jolla, CA, USA) was used for statistical analysis. Each experiment was repeated at least three times.

## Author contributions

All authors were involved in drafting the article or revising it critically for important intellectual content, and all authors approved the final version to be published. YH and HF had full access to all of the data in the study and took responsibility for the integrity of the data and the accuracy of the data analysis. YH, KL, and YP conceived and designed the project. YP, JW, YX, and DL participated in the animal experiments. YP and SZ, wrote the manuscript. HF and JZ revised it. All authors reviewed the manuscript.

### Conflict of interest statement

The authors declare that the research was conducted in the absence of any commercial or financial relationships that could be construed as a potential conflict of interest.
