# Supplementary material for: GSKJ4 Protects Mice Against Early Sepsis via Reducing Proinflammatory Factors and Up-Regulating MiR-146a
Source: Front Immunol. 2018 Oct 2;9:2272. doi: 10.3389/fimmu.2018.02272 (PMC6179039; doi:10.3389/fimmu.2018.02272)
Supplement: Supplementary file 1 [file Data_Sheet_1.docx]

# Supplementary Methods

Primers used in this study.

Primers for Chip-qPCR

| Gene | Forward | Reverse |
| --- | --- | --- |
| miR-146a | ATCCTCCTGCCTTAACCTC | CAGTGATACCTGCCATTCTC |

Primers for RT-qPCR

| Gene | Forward | Reverse |
| --- | --- | --- |
| GAPDH | TGACCTCAACTACATGGTCTACA | CTTCCCATTCTCGGCCTTG |
| IL-1β | TTCAGGCAGGCAGTATCACTC | GAAGGTCCACGGGAAAGACAC |
| KDM6A | CATAGACTTGCATCAGATCCTCC | CGGGCGGACAAAAGAAGAAC |
| KDM6B | AGTGAGGAAGCCGTATGCTG | AGCCCCATAGTTCCGTTTGTG |

## Supplementary Figures


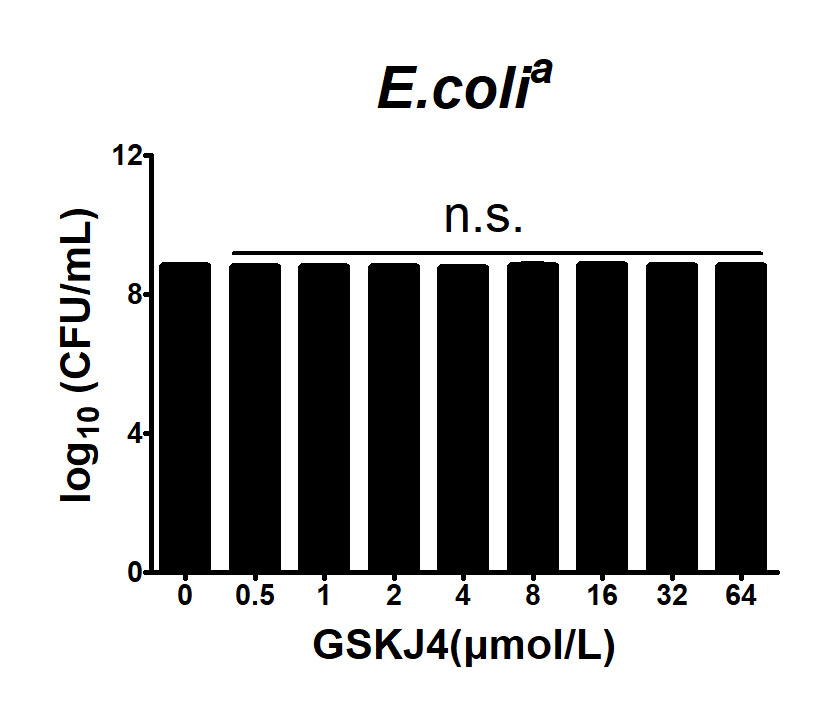


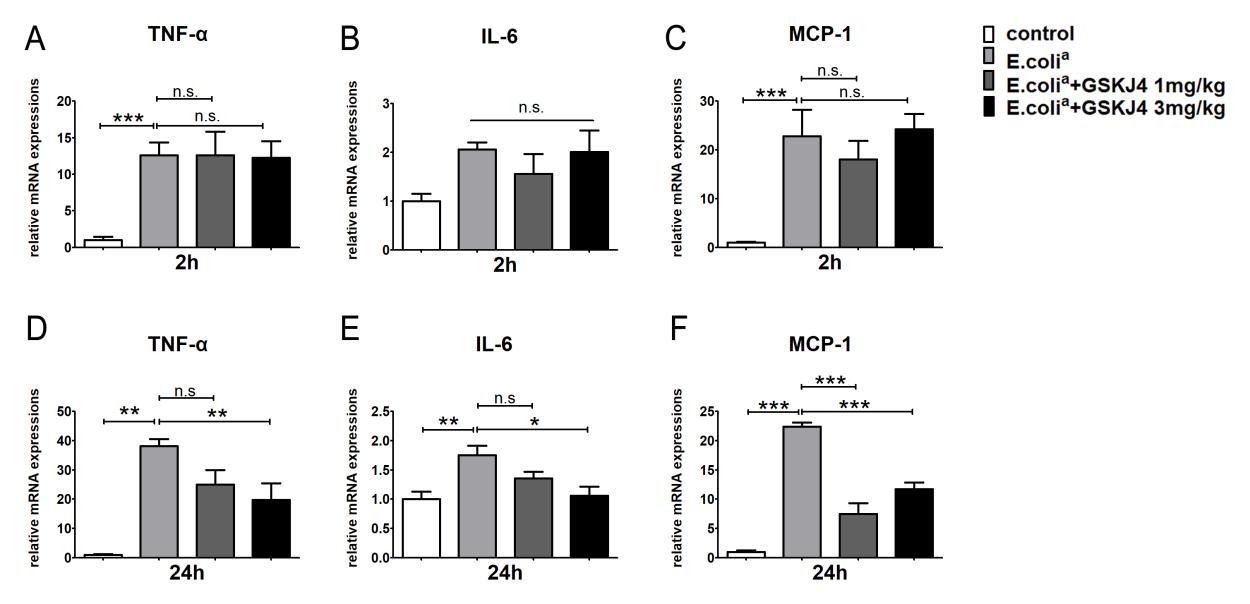


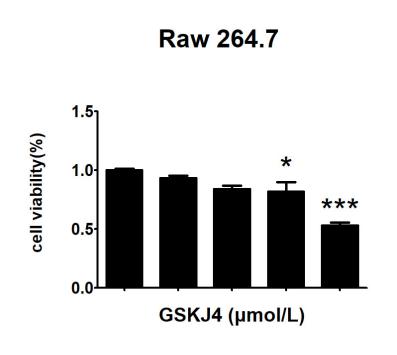


**Figure legends**

**Supplementary Figure 1. Effect of GSKJ4 on the viability of *Escherichia coli*.** Time-kill assays with GSKJ4 at different concentrations for 24 h using inocula of 1 × 10^5^ CFU/mL.

**Supplementary Figure 2.** **GSKJ4 protects mice against septic death.** ICR mice were administered intraperitoneally (i.p.) with GSKJ4 (1 or 3 mg/kg body weight) or normal saline 1h prior to bacterial infection intraperitoneally (i.p.) with viable clinical *Escherichia coli* (*E. coli^a^*) (1 × 10^7^ CFU/mouse). The mRNA expression levels of TNF-α (**A**), IL-6 (**B**) and MCP-1 (**C**) in peritoneal macrophage at 2 h post infection were determined by quantitative PCR (7 mice per group). The mRNA expression levels of TNF-α (**D**), IL-6 (**E**) and MCP-1 (**F**) in peritoneal macrophage at 24 h post infection were determined by quantitative PCR. Data are shown as mean ± SEM (n = 7). *P < 0.05; **P < 0.01; ***P < 0.001.

**Supplementary Figure 3. Effect of GSKJ4 on the viability of macrophages.** Raw264.7 cells were treated with GSKJ4 at different concentrations for 24 h. Cell viability was determined by CCK-8 assay. Data are shown as mean ± SEM (n = 7). *P < 0.05; **P < 0.01; ***P < 0.001.
